# Clinical evaluation of a multiplex droplet digital PCR for pathogen detection in critically ill COVID-19 patients with bloodstream infections

**DOI:** 10.1007/s15010-023-02157-x

**Published:** 2023-12-21

**Authors:** Yanbing Li, Kangkang Huang, Jun Yin, Zheren Tan, Manli Zhou, Jiaoyang Dai, Bin Yi

**Affiliations:** 1 Department of Laboratory Medicine, Xiangya Hospital, Central South University, Changsha, Hunan 410008 People’s Republic of China; 2 National Clinical Research Center for Geriatric Disorders, Xiangya Hospital, Central South University, Changsha, 410008 Hunan People’s Republic of China; 3 Department of Neurology, Xiangya Hospital, Central South University, Changsha, 410008 Hunan People’s Republic of China; 4 Intensive Care Unit, Xiangya Hospital, Central South University, Changsha, 410008 Hunan People’s Republic of China; 5 Department of Laboratory Medicine, Xiangya Medical School, Central South University, Changsha, Hunan 410008 People’s Republic of China

**Keywords:** Multiplex droplet digital PCR, Bloodstream infections, Severe Acute Respiratory Syndrome Coronavirus 2, Pathogen detection, Antimicrobial resistance

## Abstract

**Background:**

Nosocomial bloodstream infections (nBSI) have emerged as a clinical concern for physicians treating COVID-19 patients. In this study, we aimed to evaluate the effectiveness of a multiplex ddPCR in detecting bacterial pathogens in the blood of COVID-19 critically ill patients.

**Methods:**

This prospective diagnostic study included RT-PCR-confirmed COVID-19 patients admitted to our hospital from December 2022 to February 2023. A multiplex ddPCR assay was used to detect common bacterial pathogens and AMR genes in blood samples of the patients, along with antimicrobial susceptibility testing (AST). The diagnostic performance of the ddPCR assay was evaluated by comparing the results with those obtained through blood culture and clinical diagnosis. Additionally, the ability of ddPCR in detecting bacterial resistance was compared with the AST results.

**Results:**

Of the 200 blood samples collected from 184 patients, 45 (22.5%) were positive using blood culture, while 113 (56.5%) were positive for bacterial targets using the ddPCR assay. The ddPCR assay outperformed blood culture in pathogen detection rate, mixed infection detection rate, and fungal detection rate. *Acinetobacter baumannii* and *Klebsiella pneumoniae* were the most commonly detected pathogens in COVID-19 critically ill patients, followed by *Enterococcus* and *Streptococcus*. Compared to blood culture, ddPCR achieved a sensitivity of 75.5%, specificity of 51.0%, PPV of 30.9%, and NPV of 87.8%, respectively. However, there were significant differences in sensitivity among different bacterial species, where Gram-negative bacteria have the highest sensitivity of 90.3%. When evaluated on the ground of clinical diagnosis, the sensitivity, specificity, PPV and NPV of ddPCR were 78.1%, 90.5%, 94.7%, and 65.5%, respectively. In addition, the ddPCR assay detected 23 cases of *bla*_*KPC*_, which shown a better consistent with clinical test results than other detected AMR genes. Compared to *bla*_*KPC*_, there were few other AMR genes detected, indicating that the application of other AMR gene detection in the COVID-19 critically ill patients was limited.

**Conclusion:**

The multiplex ddPCR assay had a significantly higher pathogen detection positivity than the blood culture, which could be an effective diagnostic tool for BSIs in COVID-19 patients and to improve patient outcomes and reduce the burden of sepsis on the healthcare system, though there is room for optimization of the panels used.- Adjusting the targets to include *E. faecalis* and *E. faecium* as well as *Candida albicans* and *Candida glabrata* could improve the ddPCR' s effectiveness. However, further research is needed to explore the potential of ddPCR in predicting bacterial resistance through AMR gene detection.

**Supplementary Information:**

The online version contains supplementary material available at 10.1007/s15010-023-02157-x.

## Background

The rapid global spread of the Omicron variant of Severe Acute Respiratory Syndrome Coronavirus 2 (SARS-CoV-2) since November 2021 has made it the dominant strain worldwide by February 2022, causing nearly 300 million infections [1]. Characterized by over 60 non-synonymous mutations, this variant has led to enhanced transmissibility, reduced virulence, and milder symptoms compared to other variants of concern (VOCs). Although patients infected with the Omicron variant have shown lower hospitalization or ICU admission rates, shorter rehabilitation times, and lower mortality rates than those infected with the SARS-CoV-2 ancestral strain or other VOCs, such as Delta, it is crucial to recognize that the severity and fatality risks should not be underestimated. Hong Kong reported 9,148 COVID-19 related deaths in May 2022 [2], and approximately 2400 patients with COVID-19 died daily in the United States in February 2022 [3]. The pandemic wave of the Omicron variant poses higher risks of severe cases and fatality to vulnerable populations, including the elderly, children, neoplasm patients, transplantation recipients, and those with compromised immunity due to comorbidities. Therefore, it is necessary to implement appropriate measures to protect these high-risk groups from infection, as well as to minimize morbidity and mortality, especially in those early identified as critically ill patients with complications.

nBSI has raised significant global health concern, particularly in critically ill patients, as it brings high morbidity and mortality. Bacterial co-infection is considered to be the primary culprit of morbidity and mortality in the context of respiratory viral infections. Recent evidence suggests that bacterial co-infection in COVID-19 may contribute to overall severity and mortality [4], which has not validated by large-scale multicenter studies so far.

Rapid detection of pathogens is crucial for early diagnosis of bloodstream infections and appropriate antibiotic administration. Currently, blood culture (BC) is the conventional but standard method for causative pathogen identification and antimicrobial susceptibility testing (AST) in the diagnosis of BSIs. However, this method is limited by suboptimal sensitivity, ranging from ≤ 10% to about 50% in patients with suspected bacteremia, febrile neutropenia, or sepsis/septic shock [5]. In COVID-19 critically ill patients, the positivity of pathogen detection using blood culture is 10–28% [6]. Due to the limited sensitivity of blood culture and weak ability to detect multiple infections, our understanding of the causative pathogens of BSI in COVID-19 patients is limited. Therefore, more researches based on molecular detection are needed. In recent years, the development of molecular detection technologies has revolutionized the field of infectious disease diagnosis. Molecular detection directly targets the nucleic acid material in patient samples, significantly reducing the time required for detection. Among these technologies, digital PCR (dPCR) has emerged as a promising tool for molecular detection of bloodstream infections. Digital PCR is called the third generation of PCR, which disperses the diluted sample solution into a large number of independent reaction units, each with one or no nucleic acid molecule. After several cycles of amplification, if a nucleic acid molecule is allocated to a reaction unit, it can be detected by fluorescence reaction and defined as a positive reaction unit. Conversely, if no nucleic acid molecule is allocated to a reaction unit, no fluorescence reaction will occur, and it will not be detected, which is called a negative reaction unit. After amplification, the positive and negative reaction units are counted, and absolute quantification of the nucleic acid molecules in the target samples can be achieved by combining Poisson distribution correction. Since each nucleic acid molecule exists in a relatively independent reaction unit and does not interfere with each other, the tolerance of PCR reaction inhibitors is greatly improved, and the impact on reaction amplification efficiency is reduced, theoretically making this technology more sensitive than real-time fluorescence quantitative PCR. On the other hand, dPCR is able to absolutely quantifies the target sample's copy number (concentration) at the endpoint without establishing a standard curve. With the unique technical advantages, dPCR has been widely used in rare mutation gene detection [7], agriculture and environmental monitoring [8, 9], and microbial detection, such as covalently closed circular DNA (cccDNA) of hepatitis B virus [10], the H275Y single nucleotide mutation of human influenza virus H1N1 [11], novel coronavirus [12, 13], mycobacterium tuberculosis [14], and biothreat pathogens [15]. Relevant literatures have demonstrated its superiority, particularly in virus and bacteria detection [16]. When compared to conventional blood culture, dPCR offers higher sensitivity, specificity, and faster turnaround times, enabling early diagnosis and timely targeted treatment for patients.

In this study, we used the multiple droplet digital PCR (ddPCR) detection system targeting 18 most common pathogens and the related seven AMR genes to detect pathogens in critically ill patients with COVID-19 bloodstream infections, and conducted a clinical evaluation based on the systematic evaluation method of ddPCR in bloodstream infections recommend by Jing Wu et al. [17]. To our knowledge, this is the first evaluation of ddPCR for pathogen detection in COVID-19 BSI patients.

## Methods

### Study design and subjects

This study was a prospective pilot diagnostic study to clinically validate the multiplex ddPCR panels for rapid detection of bacterial pathogens in suspected BSIs of critically COVID-19 ill patients. This work was performed in Xiangya Hospital of Central south university, a large-scale tertiary care hospital, from December 2022 to February, 2023. The RT-PCR confirmed COVID-19 patients admitted to our hospital with suspected BSIs were eligible and consecutively recruited for the study. Patients with age < 18 years or mental disease or pregnant women were excluded from the study. Contaminated or damaged samples were also eliminated. For patients with suspected BSI, if both BC and ddPCR tests are negative, the study protocol allowed them to continue participating in the testing. However, patients with initial positive results of BC or ddPCR tests are not suggested to repeat the ddPCR testing within the following 7 days, except a new BSI attack is considered. Clinical data were extracted from the hospital’s electronic medical record, including the demographic, comorbidities, surgical intervention, organ dysfunction, and clinical outcomes. Severity of disease was assessed by the Acute Physiology and Chronic Health Evaluation II (APACHE II) scoring system. This study was approved by the Institution Review Board and Ethics Committee of Central South University.

### Sample collection for BC and ddPCR testing

For COVID-19 patients with suspected concurrent BSIs in clinical practice, blood was collected from both sides of the upper extremities into two bottles (one for aerobic culture, and the other, anaerobic), with 10–20 mL of blood in each bottle. Additionally, 2.5–3 mL of whole blood using EDTA anticoagulant was collected. The blood cultures were incubated for a maximum of 5 days (using BD BACTEC FX; BD Biosciences) and any pathogens in positive cultures were identified using matrix-assisted laser desorption/ionization-time of flight mass spectrometry (MALDI-TOF MS) (MALDI-TOF MS; Bruker Daltonik GmbH, Bremen, Germany). Coagulase-negative staphylococci (CoNS) were deemed clinically relevant only when detected in more than 50% of all blood culture sets collected from a patient on the same day; otherwise, CoNSs were considered indicative of potential contamination during the pre-analytical phase. The isolates were then tested for antimicrobial susceptibility using the Kirby-Bauer disk diffusion method and VITEK^®^ 2 COMPACT, and the results were interpreted according to the Clinical and Laboratory Standards Institute guidelines (M100-ED30) [18]. The results of conventional culture were analyzed for pathogen detection and resistance patterns. Strains that showed resistance to imipenem or meropenem were identified as carbapenem-resistant; modified carbapenem inactivation method (mCIM) and EDTA-carbapenem inactivation method (eCIM) for phenotyping were used to test the serine carbapenemase (SCARB) and the metallo-*β*-lactamase (MBL) of the carbapenem-resistant *Enterobacteriaceae* (CRE) according to M100-ED3018, and PCRs were performed to detect carbapenem-encoding resistance genes (*bla*_*KPC*_ and *bla*_*NDM*_) as described previously [19]. Methicillin-resistant *Staphylococcus sp*. was regarded to be *mecA* positive [18].

### Plasma DNA extraction and ddPCR testing

The multiplex ddPCR testing platform (Pilot Gene Technologies. Hangzhou, China) has six fluorescence channels to read the detection chip and five panels for each sample. Each panel is a multiple PCR reaction system. The five panels cover five detection systems which can be used simultaneously or separately, including detection system 1, which targets *Pseudomonas aeruginosa*, *Klebsiella pneumoniae*, *Escherichia coli,* and *Acinetobacter baumannii*; detection system 2, which targets *Staphylococcus aureus*, *Enterococcus spp*., *Streptococcus spp*., and *Candida spp*.; detection system 3, which targets *Coagulase-negative Staphylococcus*, *Burkholderia cepacia*, *Stenotrophomonas maltophilia, serratia marcescens, and Proteus mirabilis*; detection system 4, which targets *Enterobacter cloacae, Citrobacter freundii, Salmonella spp., Bacteroides fragilis and Morganella morganii*; and detection system 5, which targets *bla*_*KPC*_, *bla*_*NDM*_ and *bla*_*IMP*_, *Oxa-48*, *mecA*, *VanA,* and *VanM* antimicrobial resistance (AMR) genes. According to the statement stated in the manufacture’s insert as a validation study, the detection sensitivity of this multiplex ddPCR testing platform is 50 copy units per mL (copies/mL) with an exception of *bla*_*KPC*_ (80 copies/mL).

The testing procedures followed the manufacturer's protocol while with some improvements. In briefly, the samples were processed into plasma by centrifugation at 1200 × *g* for 15 min, taking approximately 25 min for sample preparation. The reaction mixture was passed through a micro-channel (Droplet Generator DG32) to generate tens of thousands of water-in-oil emulsion droplets within 20 min. After PCR amplification for 50 min by Thermal Cycler TC1, droplet counts and amplitudes were scanned and analyzed within 30 min using a chip scanner CS5 and Gene PMS software (v1.0.4.220303). Positive controls were synthesized DNA fragments, while DNase free water or blood samples from three healthy subjects were used as negative controls. The ddPCR results reported the copies of each targeted pathogen or gene. The testing process took no more than 2.5 h in total.

### Definition and interpretation of BSI and ddPCR results

Two trained physicians independently verified the results of both ddPCR and BCs. Results from BCs involving targeted pathogens or AMR genes in the ddPCR were summarized for further analysis of BSI and ddPCR results. Polymicrobial infection was defined as an episode in which more than one microorganism was detected by either ddPCR or blood culture. Culture-proven BSI was defined as positive blood cultures in a patient with systemic signs of infection, which may be secondary to a documented source or primary, according to the definitions released by the National Healthcare Safety Network [20]. Suspected infectious episodes of all routine microbiological cultures were collected within 7 days of enrollment according to the standard microbiology laboratory procedures. A composite clinical infection standard was defined, consisting of all microbiological results plus clinical adjudication [21, 22]. A positive ddPCR result indicated the presence of one or more target bacteria, while a negative result indicated the absence of any target bacteria. The BSI and ddPCR results were classified as concordant (both positive and negative) or discordant. Cases in which BCs were positive but ddPCR results were negative or different were defined as presumptive false-negative cases. To resolve discrepancies, discordant ddPCR + /BSI − results were classified as probable BSI, possible BSI, or presumptive false-positive cases [21, 22]. The following definitions were used for each classification: (i) probable: ddPCR result was concordant with a microbiological test performed within seven days of sample collection from another extra-blood site; (ii) possible: without microbiological data, but ddPCR result had potential for pathogenicity based on clinical presentation and laboratory findings; (iii) presumptive false-positive: ddPCR result was inconsistent with clinical presentation.

### Statistical analysis

The median and interquartile range (IQR) were used to express continuous variables, while frequencies and percentages were used to report categorical variables. Differences in sensitivity, specificity, and positive and negative predictive values between BCs and ddPCRs were assessed using the Chi-square test. The primary outcomes were the sensitivity and specificity of ddPCR testing, which were determined by comparing positive BC results with ddPCR-targeted pathogens and AMR genes. The secondary outcomes were the clinical validation of ddPCR testing for diagnosing suspected BSIs, which were compared with all microbiological cultures and the composite clinical diagnosis. Per-assay calculations were performed by analyzing results for individual pathogens in each sample separately. Statistical analyses were conducted using IBM SPSS Statistics software (v 23.0) (IBM, Armonk, NY, USA), with a *P* value of less than 0.05 considered statistically significant.

### Ethical statement

The protocol was approved by the Ethical Committee of the Xiangya Hospital of Central South University (no. 202308645). All the clinical samples included in this study were part of the routine hospital laboratory procedure. All participants gave a written informed consent prior to their inclusion in the study.

## Results

### Clinic characteristics

This study investigated the co-detection of COVID-19 and BSIs in 200 samples obtained from 184 critically ill patients suspected to have COVID-19 infection. Both BC and ddPCR methods were used. Of the 15 patients with negative test results at first, but BSI diagnosis was still considered by the physicians, samples were additionally collected for a second test, and one patient was up to be tested for three times. Table 1 presents the clinical characteristics of the patients. The average age of the patients was 72.0 years (range 59–82), with males accounting for 76.5%. The most common comorbidities were hypertension (53.3%), hypoproteinemia (46.7%), heart disease (41.3%), anemia (39.7%), and diabetes mellitus (37.5%). The mean APACHE II score was 26 points. Among these patients, 65.2% received invasive mechanical ventilation, approximately half (51.6%) were treated with vasopressors, and 53.8% experienced treatment failure.

**Table 1:** Clinical characteristics of the critically ill patients with COVID-19

Clinical characteristics	*N* = 184
Age, years, [median (IQR)]	72 (59–82)
Male, *n* (%)	137 (76.5)
Comorbidities	
Surgery performed before 14 days of inclusion, *n* (%)	66 (35.9)
Hypertension, *n* (%)	98 (53.3)
Diabetes mellitus, *n* (%)	69 (37.5)
Heart Disease, *n* (%)	76 (41.3)
Stroke, *n* (%)	14 (7.6)
CKD, *n* (%)	37 (20.1)
Malignant tumor, *n* (%)	12 (6.5)
COPD, *n* (%)	12 (6.5)
Immunosuppressive, *n* (%)	9 (4.9)
Hepatobiliary diseases, *n* (%)	39 (21.2)
Anemia, *n* (%)	73 (39.7)
Hypoproteinemia, *n* (%)	86 (46.7)
Transplant, *n* (%)	9 (4.9)
Stress gastrointestinal bleeding, *n* (%)	14 (7.6)
VMC (Viral myocarditis), *n* (%)	4 (2.2)
Invasive mechanical ventilation, *n* (%)	120 (65.2)
Treated with vasopressors, *n* (%)	95 (51.6)
APACHE II score, [median (IQR)]	26 (16–36.3)
Treatment failure, *n* (%)	99 (53.8)

*IQR*, interquartile range; *CKD*, chronic kidney disease; *COPD*, chronic obstructive pulmonary disease; *VMC*, Viral myocarditis; *APACHE II*, Acute Physiology and Chronic Health Evaluation II

### Performance of blood culture

**Fig. 1:**
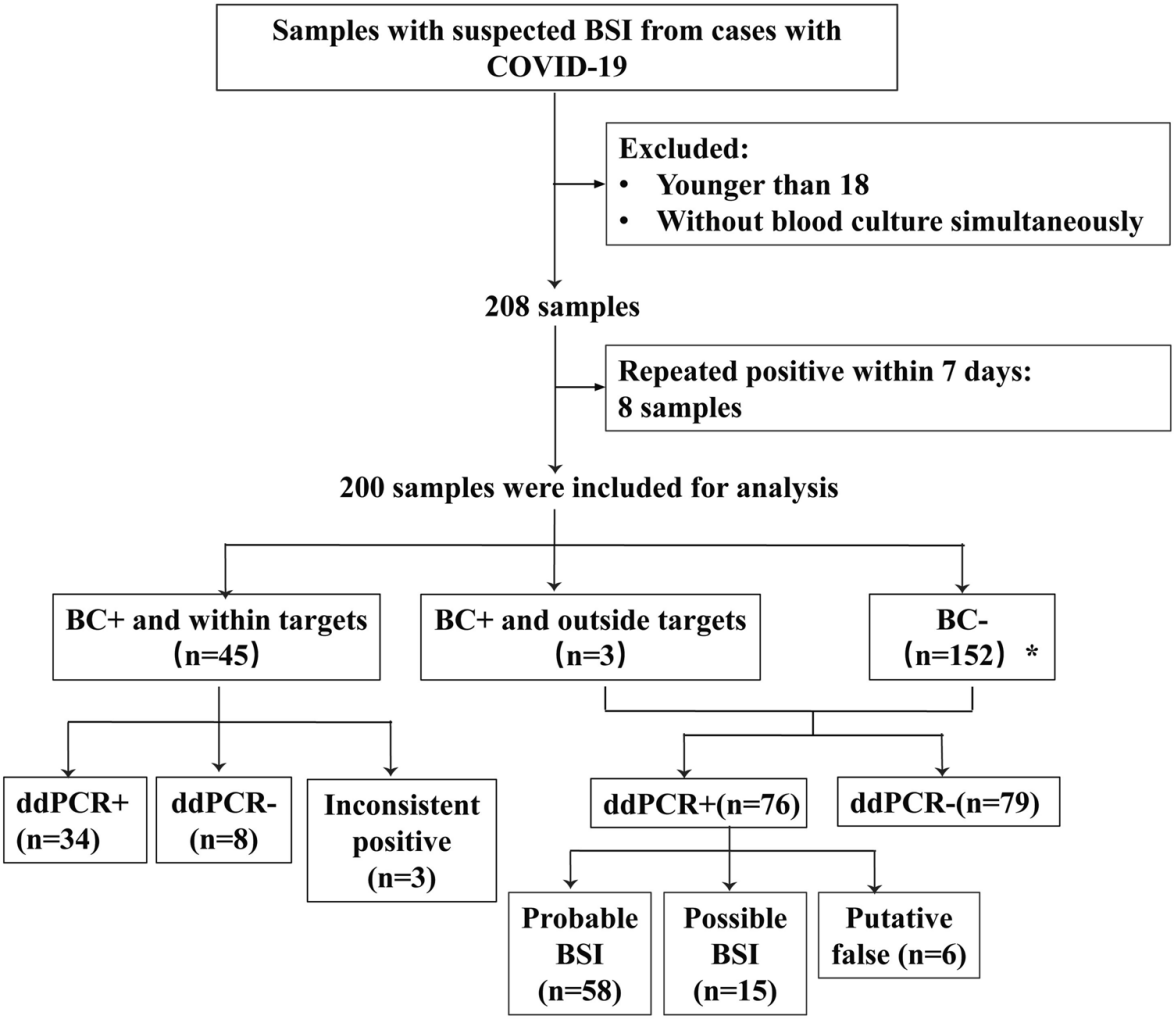
Flow-chart for patient enrollment and results analysis. *Including 3 samples judged as contaminated for isolated CoNS while present < 50% of all blood culture sets

**Fig. 2:**
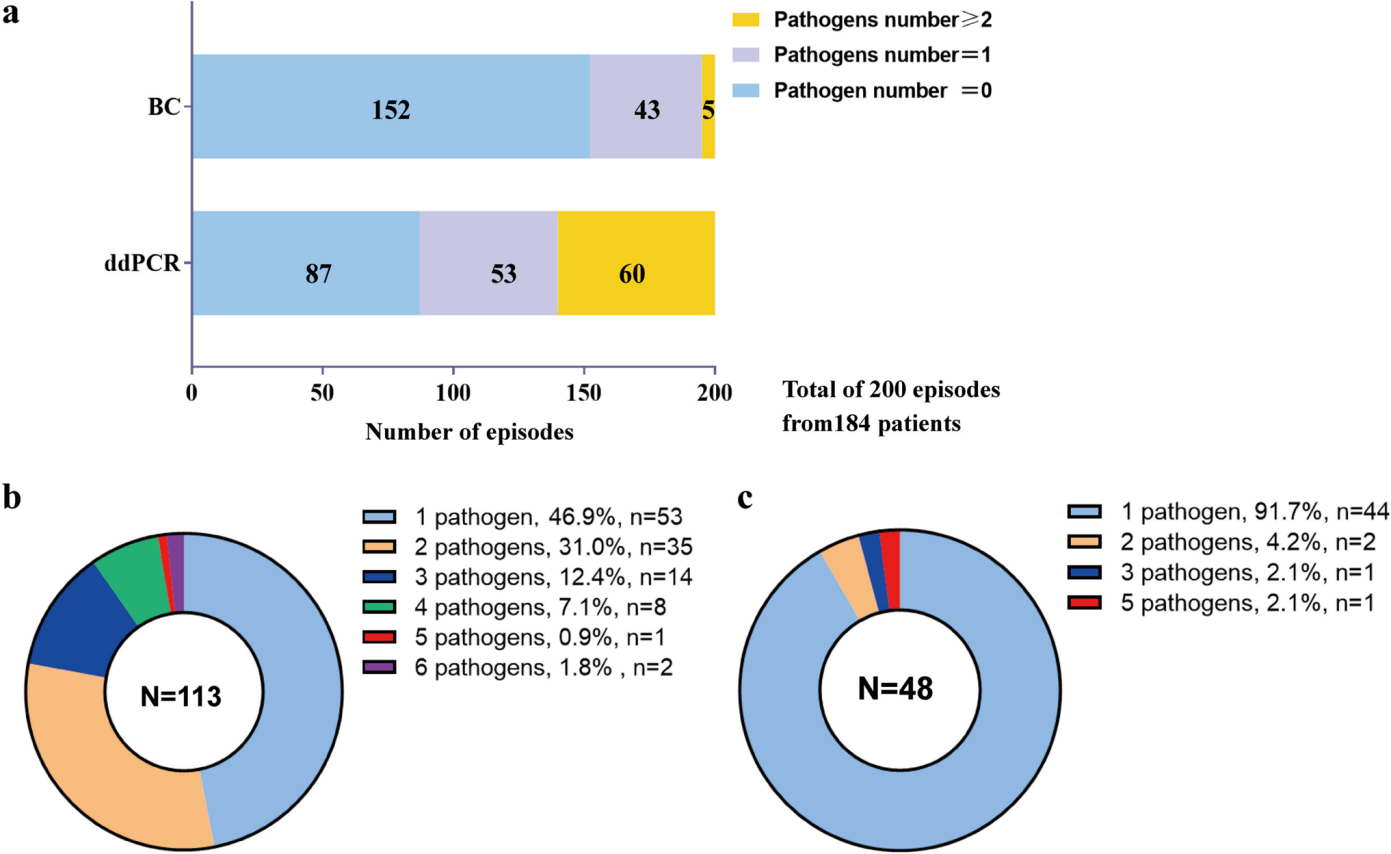
Pathogens detected by ddPCR and blood culture method. **a** Detection number of pathogens were compared between ddPCR and BC; Counts and percentage of co-infection in patients with ddPCR-positive (**b**) and blood culture-positive results (**c**)

**Fig. 3:**
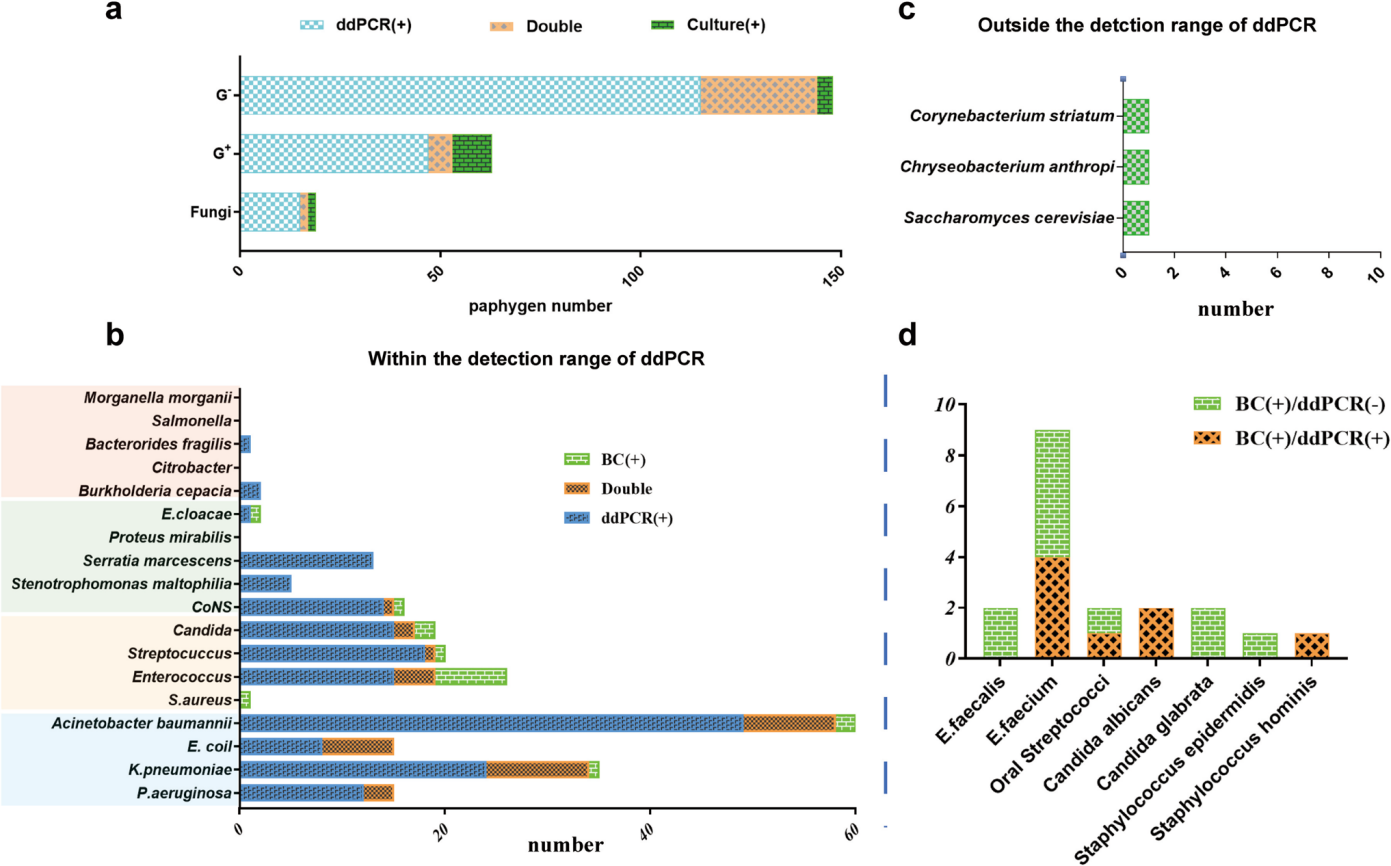
Comparision analysis of pathogens detected by ddPCR and BC method. **a** Categorization of infection events detected by ddPCR and BC method alone or simultaneously. Distribution of pathogens detected by ddPCR and BC within (**b**) and outside **c** the range of ddPCR-targeted organisms. **d** Within ddPCR-targeted organisms and detected to species level by BC method. **b** The bacteria in the blue, orange, green, and red modules are distributed in panel 1, 2, 3, and 4, respectively.

From a total of 200 samples obtained from 184 patients, 48 (24.0%) were positive for blood culture (Fig. 1). Among them, 44 samples were positive on the initial testing, while the remaining four samples were positive when repeating the test in 15 patients: 1 patient showed positivity only on the second testing with BC method, and 3 patients showed positivity with both the BC and ddPCR methods. single or multiple bacteria detected among the 48 cultured positive samples were shown in Fig. 2c. and a total of 56 bacterial strains were obtained from the 48 positive samples, comprising 17 g-positive strains (30.4%), 34 g-negative strains (60.7%), and 5 fungal strains (8.9%) as shown in Fig. 3a. Of the 45 samples that tested positive within the ddPCR targets, 53 pathogens were identified and their distribution were shown in Fig. 3b. In addition, three BC-positive strains (1 C*hryseobacterium anthropic*, 1 *Corynebacterium striatum*, and 1 *Saccharomyces cerevisiae*) which were usually associated with opportunistic infections in immunocompromised patients, were not included in our ddPCR panels (Fig. 3c), due to the limited number of targets introduced by the multiplex ddPCR assay.

### Performance of ddPCR

Out of 200 samples obtained from 184 patients, ddPCR showed positivity in 113 (56.5%) cases (Fig. 1). Among them, 101 cases (50.5%) were tested positive on initial testing, while 11 cases (5.5%) on the second, and 1 case (0.5%), the third. Of the 113 positive cases, 53 (46.9%) were detected with single bacteria, while 60 (53.1%), with multiple bacteria (Fig. 2a). The positivity rate for detecting multiple pathogens was significantly higher by ddPCR than using BC method (*p* < 0.001) (Table 2). 2–6 pathogens were detected in the 60 samples that with multiple bacteria (Fig. 2b). A total of 214 pathogens were detected in the 113 ddPCR-positive cases, of which, 144 (67.3%) were gram-negative bacteria; of them the top two strains were *A. baumannii* (*n* = 58) and *K. pneumoniae* (*n* = 34). Additionally, 53 (24.8%) Gram-positive pathogens were detected by ddPCR, including *Enterococcus* (*n* = 19), *Streptococcus* (*n* = 19), and CoNS (*n* = 15). Furthermore, ddPCR detected fungi in the remaining 17 strains (7.9%), which were identified at the genus level as Candida. Positive tests in Panel 1, Panel 2, Panel 3, and Panel 4 accounted for 122 cases (57.0%),,55 cases (25.7%),,34 (15.9%) and 3 (1.4%) cases, respectively (Fig. 3b). Out of the 17 strains of Candida detected by ddPCR, 5 samples (29.4%) tested positive for a single bacterium, whereas 12 samples (70.6%) showed the presence of multiple bacteria.

**Table 2:** Comparison of pathogen numbers detected by ddPCR and blood culture method

Number of pathogens	ddPCR	BC	*P* value
0	87	152	< 0.001
1	53	44	0.292
≥ 2	60	4	< 0.001

### Comparison analysis between ddPCR and blood culture

In 200 specimens obtained from 184 patients, ddPCR and conventional blood culture were used in combination to detect 121 (60.50%) infection events with a total of 233 pathogens. Among 15 patients who underwent repeated testing, three were positive by both the BC method and ddPCR, nine were positive only by ddPCR, and two remained negative even after repeated testing. One of the two patients had a blood sample tested using mNGS, and the result revealed mycobacterium tuberculosis positivity and so the clinical diagnosed to be pulmonary tuberculosis. Of the 121 infection events detected, 53.10% (60/113) were mixed infections identified by ddPCR alone, while 9.09% (4/44) were identified by BC. Most infection events were identified by ddPCR alone (76/184) or by both ddPCR and conventional blood culture (34/184). Additionally, while there were three samples that were positive by both BC and ddPCR, but presented with different bacterial species (Fig. 1). That was *E. faecium, A. baumannii* and *oral streptococci* detected by BC in three patients respectively, but *A. baumannii, mixed infection (K. pneumoniae*, *Streptococcus*, *Enterococcus* and *Candida) and another mixed infection* (*A. baumannii*, *P. aeruginosa*, and *Serratia marcescens*) was detected by ddPCR. We classified these three samples as presumable false-negative results and put them in subsequent ddPCR studies for pathogen evaluation. Based on the detection results of the 230 pathogens within ddPCR targets, we analyzed the performance of ddPCR versus BC. Comparing ddPCR to BC, ddPCR alone was positive for 177 pathogens, ddPCR and BC were both positive for 37 pathogens, and BC alone was positive for 16 pathogens (Fig. 3a, b). For CoNS, *Enterococcus*, *Streptococcus*, and Candida at the genus level identified by ddPCR, BC verified to the species level (Fig. 3d). Among the 16 pathogens that were only positive by BC, there were 7 strains of *Enterococcus* (2 *E. faecalis* and 5 *E. faecium*), and only one or two strains are present in other bacteria species. This suggests that the *Enterococcus* detection system via ddPCR requires optimization. When comparing the 177 single positive pathogens identified by ddPCR with test results from other samples, we have cultured 95 similar strains in other samples (Table S2). As assessing ddPCR versus all culture results. The overall sensitivity, specificity, positive predictive value (PPV), and negative predictive value (NPV) for ddPCR were 77.3%, 74.1%, 81.4%, and 69.0%, respectively (Table 3). While the sensitivity of ddPCR was higher for Gram-negative bacteria (90.3%) compared to Gram-positive bacteria (50.0%) and fungi (75.0%).

**Table 3:** Positive and negative agreement of ddPCR and BC, all microbiological testing within the detection range of ddPCR

Sample (*n* = 200)		ddPCR ( +)	ddPCR (−)	Sensitivity (%, 95% CI)	Specificity (%, 95% CI)	PPV (%, 95% CI)	NPV (%, 95% CI)
Total	BC+	34*	11	75.6 (61.3–85.8)	51.0 (43.2–58.7)	30.9 (23.0–40.1)	87.8 (79.4–93.0)
	BC−	76	79				
G−	BC+	28	3	90.3 (75.1–96.7)	65.1 (57.6–71.9)	32.2 (23.3–42.6)	97.4 (92.5–99.3)
	BC-	59	110				
G +	BC+	7	7	50.0 (26.8–73.2)	83.3 (77.2–88.1)	18.9 (9.5–34.2)	95.5 (91.1–97.8)
	BC−	30	156				
Fungi	BC+	3	1	75.0 (30.1–98.7)	95.4 (91.5–97.6)	25.0 (8.9–53.2)	99.5 (97.1–99.9)
	BC−	9	187				
Positive by all microbiological testing		92	27	77.3 (69.0–83.9)	74.1 (63.6–82.4)	81.4 (73.3–87.5)	69.0 (58.6–77.7)
Negative by all microbiological testing		21	60				

*G−*, Gram-negative bacteria; *G+*,Gram-positive bacteria; *PPV*, positive predictive value; *NPV*, negative predictive value

*Including 4 mixed infection, 2 with a G− pathogen and a G + pathogen, 1 with two G- pathogens and 1 with a G− pathogen, a G + pathogen and a fungi

### Comparing ddPCR performance with all microbiology culture and clinical diagnosis

Of 113 ddPCR-positive cases, 76 were BC negative, and additional 3 were ddPCR + /BC+ but with inconsistent bacteria. Further analysis was necessary for these 79 cases by combining the culture results of all samples from other parts of the body of the same patient within 7 days. Among them, pathogens detected by ddPCR had partial or complete matches with isolates from other parts in some episodes, including four instances of poly-pathogen infections detected by ddPCR, which were also isolated in other parts, and 23 poly-pathogen infections partially isolated from other parts (Table S1). In accordance with the definitions of probable BSI and possible BSI outlined in the methods section, also, the 79 inconsistent test results were subjected to detailed analysis by integrating both clinical and composite microbiological evidence. The results indicated that out of the aforementioned 79 episodes, 48 (60.8%) fulfilled the criteria for probable BSI, while 13 (16.5%) were categorized as possible BSI. The remaining 18 cases (22.8%) were presumptive false positives, as illustrated in Fig. 1. Furthermore, when analyzing the sensitivity and specificity of ddPCR testing for identifying probable BSIs across all microbiological testing and clinical diagnoses, it was found that the sensitivity and specificity values were 78.1% and 90.5%, respectively, with PPV and NPV of 94.7% and 65.5%, respectively, though the obtained sensitivities displayed a high degree of variability between individual detection panels (Table 4). Additionally, we noticed that broad-spectrum antibiotics such as Piperacillin tazobactam, Meropenem were the most frequently empirical antibiotics drug in clinical. In cases of ddPCR-positive but BC-negative episodes, 36.7% (29/79) of patients received targeted antimicrobial therapy, 30.4% (24/79) received partial targeted treatment due to polymicrobial infections, and 32.9% (26/79) did not receive any appropriate treatment prior to the BC and ddPCR tests. These initial findings suggest that ddPCR has the potential to rapidly identify specific pathogens.

**Table 4:** Positive and negative agreement of ddPCR and clinical diagnosis within the detection range of ddPCR

Sample (*n* = 200)		Positive by clinical diagnosis	Negative by clinical diagnosis	Sensitivity (%, 95% CI)	Specificity (%, 95% CI)	PPV (%, 95% CI)	NPV (%, 95% CI)
Total	ddPCR ( +)	107	6	78.1 (70.5–84.2)	90.5 (80.7–95.6)	94.7 (88.9–97.5)	65.5 (55.1–74.7)
	ddPCR (−)	30	57				
Panel 1	ddPCR ( +)	84	2	61.3 (53.0–69.1)	96.8 (89.1–99.4)	97.7 (91.9–99.6)	53.5 (44.4–62.4)
	ddPCR (−)	53	61				
Panel 2	ddPCR ( +)	39	3	28.5 (21.6–36.5)	95.2 (86.9–98.7)	92.9 (81.0–97.5)	38.0 (30.8–45.7)
	ddPCR (−)	98	60				
Panel 3	ddPCR (+)	29	2	21.2 (15.2–28.7)	96.8 (89.1–99.4)	93.6 (79.3–98.9)	36.1 (29.2–43.6)
	ddPCR (−)	108	61				
Panel 4	ddPCR (+)	3	0	2.2 (0.6–6.2)	100 (94.3–100)	100 (43.9–100)	32.0 (25.9–38.8)
	ddPCR (−)	134	63				

### Evaluation of the AMR genes detected by ddPCR

Our findings indicated the presence of 23 episodes that tested positive for *bla*_*KPC*_ using ddPCR. Among these cases, *K. pneumoniae* and the *bla*_*KPC*_ gene co-occurred in 21 (91.3%) cases, and this finding was particularly relevant to the clinical context (Table 5). In comparison to blood culture (BC) results, 10 cases of BC-positive *K. pneumoniae* exhibited resistance to carbapenems, including eight strains that showed SCARB expression and two strains that expressed MBL. Of the eight SCARB-producing strains, seven had detectable *bla*_*KPC*_ genes (7/8); however, among the two MBL-producing strains, the *bla*_*KPC*_ gene was not detected. 16 out of the 23 episodes that were resistant to carbapenems and expressed SCARB were identified by other microbiological and antimicrobial susceptibility tests. Regarding the *bla*_*IMP*_* and bla*_*NDM*_ gene, PCR analysis for *bla*_*NDM*_ gene detection was positive in two *K. pneumoniae* strains that were identified as causative pathogens based on microbiological testing and MBL production in carbapenemase typing test. However, the ddPCR test did not produce a positive result. Except *bla*_*KPC,*_ few other AMR genes were detected, which indicate further research is needed to explore the potential of ddPCR in predicting bacterial resistance through AMR gene detection.

**Table 5:** AMR genes detected by ddPCR and the related pathogens detected by BC and all microbiological testing

AMR genes	Pathogens	ddPCR + *n* (%)	BC+ and according to AST *n*, (%)	Microbiological testing and according to AST *n*, (%)
*bla*_*kpc*_ (*n* = 23)	*K. pneumoniae*	21 (91.3)	7 (30.4)	17 (73.9)
	None	2 (8.7)	16 (69.6)	6 (26.1)
*bla*_*NDM*_/*bla*_*IMP*_ (*n* = 3)	*K. pneumoniae*	2 (66.7)	0	2 (66.7)
	None	1 (33.3)	3 (100)	1 (33.3)
*OXA*_*48*_ (*n* = 2)	CRE or CRAB	2 (100)	0	2 (100)
	None	0	2 (100)	0
*mceA* (*n* = 6)	CoNS	4 (66.7)	–	–
	*Staphylococcus hominis*	–	1 (16.7)	1 (16.7)
	None	2 (33.3)	5 (83.3)	5 (83.3)
*vanA*/*vanM* (*n* = 0)	–	–	–	–

## Discussion

Since November 2021, the Omicron variant and its subvariants of the novel coronavirus SARS-CoV-2 have been spreading widely across the globe. Compared to previous variants, the Omicron variant has lower virulence and milder symptoms following infection. However, some patients infected with Omicron can still progress to critical illness, especially elderly individuals or those with weakened immune systems. The latest predictive model suggests that age, neutrophils, lymphocytes, IL-2, IL-10, and procalcitonin are the major variables in predicting progression to severe illness, particularly white blood cell count and procalcitonin inflammatory index, which are commonly used in clinics to judge sepsis [23]. As Omicron continues to spread globally, it has been estimated that the Omicron subvariant XBB.1.9 will surpass XBB.1.16 and become the dominant strain [24, 25], resulting in an increase in COVID-19 cases in countries such as India, China, and the United States.

In COVID-19 hospitalized patients, there is an increased number of bloodstream infections (BSI) in the intensive care unit (ICU) because they require invasive devices such as central venous catheters, extracorporeal membrane oxygenation (ECMO), or renal replacement therapy [26, 27]. However, there are still few studies focusing on the detection and clinical relevance of bacteremia in these COVID-19 patients. Establishing a rapid detection method for bacterial infections in critically ill patients with SARS-CoV-2 infection has broad clinical application prospects and social value. Although current debates surround whether secondary bacterial infections affect the prognosis of SARS-CoV-2 infection, some studies suggest that bacterial infections could worsen pulmonary inflammation and increase mortality rates. Some studies found that COVID-19 patients with concomitant bacterial infections required mechanical ventilation and longer ICU stays [26, 27], while another retrospective study found that the mortality rate among COVID-19 patients with bacterial infections was significantly higher than those without bacterial infections (43.1% vs 12.3%) [28]. Therefore, early and accurate selection of antibacterial drugs is essential in controlling the spread of the pathogens.

Currently, the positivity of pathogen detection in COVID-19 critically ill patients with BSI using blood culture ranges from 10 to 28% [6]. However, these studies are often based on small sample sizes. In a study conducted in Mexico [27], common pathogens found in primary bacteremia were *Chryseobacterium indologenes*, *E. coli*, and *Streptococcus*, while *P. aeruginosa* and *Enterococcus Marina* were found in secondary bacteremia. Cuntrò et al.[29] identified *E. coli*, *K. pneumoniae*, *P. aeruginosa*, and *A. baumannii* as Gram-negative strains, and *E. faecalis*, *E. faecium*, *S. aureus*, and *S. pneumoniae* as the Gram-positive cocci responsible for COVID-19 BSI. Moreover, another study revealed that the isolated bacteria from COVID-19 BSI patients are different from non-COVID-19 BSI patients [30]. Specifically, COVID-19 patients had higher incidence rates of *Enterococcal* (20.5% vs. 9%) and *Acinetobacter spp.* (18.8% vs. 13.6%). To address this issue, we designed a multiplex droplet digital PCR (ddPCR) panel based on global and local pathogen epidemiology targeting 18 of the most common pathogens and seven AMR genes. For the first time, our study evaluated and compared the detection of pathogens responsible for COVID-19 BSI in critically ill patients using both blood culture and multiplex ddPCR methodology.

The study found that the majority of patients (*n* = 93) were between 46 and 60 years old. Blood culture results disclosed that gram-negative pathogens were predominantly identified (60.7%), followed by gram-positive pathogens (30.4%) and yeast (8.9%). Among these isolates, *K. pneumoniae*, *A. baumannii*, and *Enterococcus sp*. were the most commonly identified pathogens, each accounting for 19.6%. The vast majority of bacteria identified through blood culture positive (BC +) (91.7%) were covered by our multiplex ddPCR targets, with only three strains falling outside of the coverage area. To broaden the scope of detection, we also performed identification at the species level for some bacteria such as *Enterococcus*, *Staphylococcus*, *Streptococcus*, and *Candida*, resulting in a wide range of pathogen detection covering bacteria and fungi. The ddPCR test had significantly higher positivity rates than the blood culture method. Of the 200 blood samples, 113 were positive (61.4%) through ddPCR, including 76 cases that were blood culture negative, and a total of 214 pathogens were detected, with 67.3% being gram-negative bacteria, 24.8% being gram-positive bacteria, and 7.9% being Candidas. A combination of blood culture and ddPCR identified 230 strains of bacteria in the targets, with *A. baumannii* (*n* = 60), *K. pneumoniae* (*n* = 35), and gram-positive *Enterococcus* (*n* = 26) and *Streptococcus* (*n* = 20) comprising the most frequently detected pathogens. In addition, *Candida* (*n* = 19) was also frequently detected. These findings are significantly different from those of Jing Wu [17], who researched non-COVID-19 bloodstream infection in ICU inpatient population using ddPCR and blood culture, identifying *K. pneumoniae*, *P. aeruginosa*, and *E. faecium* as the most common pathogens.

However, 11 blood culture-positive samples within the target range tested negative by ddPCR in our study. Out of the 45 BC-positive cases detected, 16 pathogens were BC positive while ddPCR negative, including 10 g-positive strains (2 *E. faecalis*, 5 *E. faecium*, 1 *Oral Streptococci*, 1 *S. aureus*, and 1 *S. epidermidis*), 4 g-positive strains (2 *A. baumannii*, 1 K*. pneumoniae*, and 1 *E. cloacae*), and 2 *Candida glabrata*. It is suspected that the bacteria count in the blood may be too low to be detected by PCR but can be detected by blood culture, which uses a larger volume of samples. However, the sensitivity of blood culture for gram-negative bacteria and fungi was found to be 50% and 75%, respectively, which was significantly lower than the sensitivity of 90.3% for gram-negative bacteria. This indicates that the reaction conditions for our ddPCR method are still suboptimal, especially for gram-positive bacteria. Further optimization of the ddPCR reaction system and conditions is required.

Polymicrobial bacteremia (PMB) is a frequently encountered condition where multiple microorganisms concurrently infect the bloodstream. It has been reported to comprise around 10–11% of the positive blood culture cases in recent studies[31]. Immunocompromised status, the presence of foreign objects, and recent surgical procedures increase the risk of PMB[32]. Notably, patients with PMB remain at high risk for death compared to cases of bacteremia caused by a single microorganism [32–34]. Unexpectedly, in our study, the rate of mixed infections among COVID-19 patients has been found over a half (60 out of 113 cases). In some instances, up to 6 different pathogenic species were detected in a single sample using ddPCR. Fungi positivity was also significantly increased, with *Candida* presented together with other bacterial pathogens in the mixed infections. This highlights the inadequacy of culture-based methods such as blood culture in detecting mixed bacterial and fungal infections. PMB, especially with invasive fungal diseases (IFDs), is a known area of reduced diagnostic fidelity for various pathogen detection methods, and it is still a challenge for high-quality detection requirement [35]. The ddPCR system utilized in this study exhibited a high detection rate of mixed pathogens, which may be attributed to the study population consisting of critically ill COVID-19 patients, suffering from severe lung damage, requiring mechanical ventilation, and possibly receiving extracorporeal membrane oxygenation therapy. When comparing the detection results of mixed pathogens by ddPCR with isolates from other body sites of the same patient within 7 days, we found that only 5 out of 60 mixed pathogen episodes were inconsistent (Table S1). Nonetheless, we need to be cautious to conclude that these mixed pathogens represent truly clinically determined PMB. In fact, some critically ill COVID-19 patients experienced rapid disease progression, making it difficult to allow sufficient time for clinicians to observe more data to make an informed decision. Further exploration is required to elucidate whether this ddPCR method truly provides a high detection rate of mixed infections.

This multi-ddPCR method can detect different bacteria at once, greatly increased the detection efficiency and highlighted the advantage of molecular detection that is independent of bacterial growth [36]. However, some bacterial species included in our system, such as *Salmonella*, *Citrobacter*, and *Morganella morganii*, were not detected. So when diagnosing BSI in COVID-19 patients, it may be necessary to optimize the design scheme and recommend adjustments such as identifying *E. faecalis* and *E. faecium* to the species level, and also the *Candida albicans* and *Candida glabrata* strains, because they have different AST pattern with each other.

For the no-COVID-19 BSI ICU inpatients, ddPCR displayed a sensitivity ranging from 58.8 to 86.7% and an aggregate specificity ranging from 73.5 to 92.2% [17]. Compared to blood culture, ddPCR showed similar sensitivity but lower specificity. However, ddPCR had a satisfactory extra detection rate, indicating that it was able to detect additional cases that blood culture could not. Importantly, 38.0% (76/200) of all tests had discordant results, with ddPCR positive while blood culture negative. Further review of clinical circumstances revealed that most of these cases were either probable (22.5%, 45/200) or possible (6.5%, 13/200) BSIs. When clinically diagnosed BSIs criteria were used as a comparator, the overall sensitivity and specificity of ddPCR were 78.1% and 90.5%, respectively. These values increased to 84.9% and 92.5% when clinically diagnosed BSI was used as true positive for the no-COVID-19 BSI ICU inpatients [17].

In addition, we designed an AMR genes detection channel in the ddPCR system. However, predicting bacterial resistance using resistance genes has always been controversial due to the many reasons for bacterial resistance and the lack of a one-to-one mapping between bacterial resistance and resistance genes. Among the 11 cultured strains of *K. pneumoniae*, 1 sensitive strain and 10 CRE strains were found, including 8 strains producing SCARB and 2 strains producing MBL. Seven out of eight strains producing SCARB tested positive for the *bla*_*kpc*_ gene, while the antimicrobial sensitivity strain and 2 MBL-producing strains did not have the *bla*_*kpc*_ gene detected. Therefore, the detection of the *bla*_*kpc*_ gene showed better consistency with clinical test results. Only a few other AMR genes were detected, indicating limited application of other AMR gene detection in the COVID-19 critically ill patient population. Notably, two *K. pneumoniae* strains that were blood culture positive were shown to be MBL-producing strains by carbapenemase typing, and the *bla*_*NDM*_ gene was detected by ddPCR but not by the *bla*_*IMP/NDM*_ channel in the ddPCR method. Additionally, the majority of *A. baumannii* detected using the cultivation method were multidrug-resistant strains, which was not anticipated in the design of the ddPCR-based AMR gene detection system. This finding highlights the necessity for a targeted design of a more suitable AMR gene detection model when applying ddPCR in the population of COVID-19 critically ill patients. Traditional bacterial drug sensitivity tests take longer to obtain results as they have to be performed after bacterium isolation and identification. The application of ddPCR for AMR genes in predicting bacterial drug sensitivity through resistance genes has always been a topic of discussion and needs further verification.

In recent years, there have been several successfully commercialized molecular assays that detect pathogens and AMR genes either directly from whole blood samples or positive blood cultures. Differently from BCID (Blood Culture Identification), assays performed with original blood samples offer the advantage of being independent of the time-consuming culture. Some narrow-based platforms primarily utilize multiplex PCR to determine target pathogens, while more extensive platforms combine broad-range PCR with amplicon sequencing. Although the multiplex ddPCR assay falls into the narrow-based category, it covers the majority of bacteria and yeast as target pathogens. Target pathogens in our study were built on the epidemiological analysis and 15-year blood culture data of our lab. The 18 pathogens included in the multiplex ddPCR panels covered over 80% of the identified positive isolates in our lab. Currently, this multiplex ddPCR platform is for research use only and costs approximately RMB 420–500 ($60–70) for one test, and the price is much lower than $135–175 per test for similar assays that are CE-IVD marked or FDA cleared [35, 37]. The experimental procedure for the multiplex ddPCR assay is relatively simple, with all steps performed in a pouch after reagent hydration. It can be semi-automatically operated with manual intervention or full-automatically handled, while the latter costs higher. The turnaround time from testing start to result is 2.0–2.5 h, which saves 1.0–3.0 h in contrast to 3.5–5 h for detection of target pathogens and AMR genes using multiplex real-time PCR-based methods, such as SeeGene MagicPlex^®^ Sepsis Test and Roche Lightcycler^®^ SeptiFas [38, 39]. More importantly, incorporated with droplet technology, the multiplex ddPCR is more sensitive than the real-time fluorescence quantitative PCR. This implies that the multiplex ddPCR assay possesses certain advantages; however, it has to be performed on specific and expensive instruments, and has higher environmental requirements for detection as well.

In summary, this study evaluated the detection efficacy of a multiple droplet digital PCR system for identifying pathogens in COVID-19 critical patients by comparing to conventional culture and clinical diagnosis. The multi-ddPCR method significantly improved the detection of mix pathogens and fungi, exhibited higher sensitivity, specificity, and faster turnaround times, enabling early diagnosis and timely targeted treatment for patients, especially those with sepsis. It suggests that the application of ddPCR in clinical settings has the potential to improve patient outcomes and reduce the burden of sepsis on the healthcare system.

Limitations of this study include the limited coverage of the ddPCR method compared to metagenomic next-generation sequencing (mNGS), as some bacteria were not detectable. The study was conducted at a single center, limiting its generalizability. The predicted drug sensitivity of multi-drug-resistant *A. baumannii* was suboptimal in this system. As this was an observational study without intervention treatments, the clinical benefits of ddPCR could not be accurately evaluated. The correlation between quantitative detection and disease progression remains unclear and requires further investigation.

### Supplementary Information

Below is the link to the electronic supplementary material.

Supplementary file1 (DOCX 15 kb)

Supplementary file2 (DOCX 14 kb)

## Data Availability

The raw data supporting the conclusions of this article will be made available by the authors, without undue reservation, to any qualified researcher.

## References

[1] WHO. WHO Coronavirus (COVID-19) Dashboard.

[2] Mefsin YMCD, Bond HS, Lin Y, Cheung JK, Wong JY, Ali ST, Lau EHY, Wu P, Leung GM, Cowling BJ (2022). Epidemiology of infections with SARS-CoV-2 omicron BA.2 variant, Hong Kong, January-March 2022. Emerg Infect Dis.

[3] Propelled by omicron, U.S. death toll from COVID-19 hits 900,000 Los Angeles, Portland: NewsHour. 2022.

[4] Devi P, Maurya R, Mehta P, Shamim U, Yadav A, Chattopadhyay P, Kanakan A, Khare K, Vasudevan JS, Sahni S (2022). Increased abundance of achromobacter xylosoxidans and bacillus cereus in upper airway transcriptionally active microbiome of COVID-19 mortality patients indicates role of co-infections in disease severity and outcome. Microbiol Spectr.

[5] Murray PR, Masur H (2012). Current approaches to the diagnosis of bacterial and fungal bloodstream infections in the intensive care unit. Crit Care Med.

[6] Leitl CJ, Stoll SE, Wetsch WA, Kammerer T, Mathes A, Bottiger BW, Seifert H, Dusse F (2023). Next-generation sequencing in critically Ill COVID-19 patients with suspected bloodstream infections: a retrospective cohort study. J Clin Med.

[7] Holm M, Andersson E, Osterlund E, Ovissi A, Soveri LM, Anttonen AK, Kytola S, Aittomaki K, Osterlund P, Ristimaki A (2020). Detection of KRAS mutations in liquid biopsies from metastatic colorectal cancer patients using droplet digital PCR, Idylla, and next generation sequencing. PLoS One.

[8] Hu G, Huang K, Zhou W, Wang R, Zhao W, Zou H, Li W, Wu S, Li M, Wang G (2023). Comparison of droplet digital PCR and real-time quantitative PCR for quantitative detection of the parasitic ciliate Ichthyophthirius multifiliis in the water environment. J Fish Dis.

[9] Lucansky V, Samec M, Burjanivova T, Lukacova E, Kolkova Z, Holubekova V, Turyova E, Hornakova A, Zaborsky T, Podlesniy P (2023). Comparison of the methods for isolation and detection of SARS-CoV-2 RNA in municipal wastewater. Front Public Health.

[10] Caviglia GP, Abate ML, Tandoi F, Ciancio A, Amoroso A, Salizzoni M, Saracco GM, Rizzetto M, Romagnoli R, Smedile A (2018). Quantitation of HBV cccDNA in anti-HBc-positive liver donors by droplet digital PCR: a new tool to detect occult infection. J Hepatol.

[11] Whale AS, Bushell CA, Grant PR, Cowen S, Gutierrez-Aguirre I, O'Sullivan DM, Zel J, Milavec M, Foy CA, Nastouli E (2016). Detection of rare drug resistance mutations by digital PCR in a human influenza a virus model system and clinical samples. J Clin Microbiol.

[12] Vasudevan H, Xu P, Servellita V, Miller S, Liu L, Gopez A, Chiu CY, Abate AR (2020). Digital droplet PCR accurately quantifies SARS-CoV-2 viral load from crude lysate without nucleic acid purification. medRxiv.

[13] Vasudevan HN, Xu P, Servellita V, Miller S, Liu L, Gopez A, Chiu CY, Abate AR (2021). Digital droplet PCR accurately quantifies SARS-CoV-2 viral load from crude lysate without nucleic acid purification. Sci Rep.

[14] Yang J, Han X, Liu A, Bai X, Xu C, Bao F, Feng S, Tao L, Ma M, Peng Y (2017). Use of digital droplet PCR to detect mycobacterium tuberculosis DNA in whole blood-derived DNA samples from patients with pulmonary and extrapulmonary tuberculosis. Front Cell Infect Microbiol.

[15] Du Y, Yan Z, Song K, Jin J, Xiao L, Sun Z, Tan Y, Zhang P, Du Z, Yang R (2022). Development and evaluation of a multiplex droplet digital polymerase chain reaction method for simultaneous detection of five biothreat pathogens. Front Microbiol.

[16] Pomari E, Piubelli C, Perandin F, Bisoffi Z (2019). Digital PCR: a new technology for diagnosis of parasitic infections. Clin Microbiol Infect.

[17] Wu J, Tang B, Qiu Y, Tan R, Liu J, Xia J, Zhang J, Huang J, Qu J, Sun J (2022). Clinical validation of a multiplex droplet digital PCR for diagnosing suspected bloodstream infections in ICU practice: a promising diagnostic tool. Crit Care.

[18] CLSI M100-ED30:2020 Performance Standards for Antimicrobial Susceptibility Testing, 30th edn.

[19] Poirel L, Walsh TR, Cuvillier V, Nordmann P (2011). Multiplex PCR for detection of acquired carbapenemase genes. Diagn Microbiol Infect Dis.

[20] Timsit JF, Ruppe E, Barbier F, Tabah A, Bassetti M (2020). Bloodstream infections in critically ill patients: an expert statement. Intens Care Med.

[21] Nguyen MH, Clancy CJ, Pasculle AW, Pappas PG, Alangaden G, Pankey GA, Schmitt BH, Rasool A, Weinstein MP, Widen R (2019). Performance of the T2 bacteria panel for diagnosing bloodstream infections: a diagnostic accuracy study. Ann Intern Med.

[22] Kalligeros M, Zacharioudakis IM, Tansarli GS, Tori K, Shehadeh F, Mylonakis E (2020). In-depth analysis of T2Bacteria positive results in patients with concurrent negative blood culture: a case series. BMC Infect Dis.

[23] Lu T, Man Q, Yu X, Xia S, Lu L, Jiang S, Xiong L (2023). Development and validation of a prognostic model based on immune variables to early predict severe cases of SARS-CoV-2 Omicron variant infection. Front Immunol.

[24] ChinaCDC: https://www.chinacdc.cn/jkzt/crb/zl/szkb_11803/jszl_13141/202304/t20230429_265709.html.

[25] WHO: https://www.who.int/activities/tracking-SARS-CoV-2-variants.

[26] Rawson TM, Moore LSP, Zhu N, Ranganathan N, Skolimowska K, Gilchrist M, Satta G, Cooke G, Holmes A (2020). Bacterial and fungal coinfection in individuals with coronavirus: a rapid review to support COVID-19 antimicrobial prescribing. Clin Infect Dis.

[27] Daniel CM, Alondra GB, Omar G, Daniel AZ, Pablo R, Octavio GC, Eva JH, Gloria SL, Ojino S (2021). Bacteremia in Critically Ill Patients with SARS-CoV 2 Infection. Sci Publish Group.

[28] Langford BJ, So M, Raybardhan S, Leung V, Westwood D, MacFadden DR, Soucy JR, Daneman N (2020). Bacterial co-infection and secondary infection in patients with COVID-19: a living rapid review and meta-analysis. Clin Microbiol Infect.

[29] Cuntro M, Manisco A, Guarneri D, Zuglian G, Vailati F, Passera M, Cavallini M, Raglio A, Farina C (2021). Blood stream infections during the first wave of COVID-19. A short microbiological retrospective picture at Papa Giovanni XXIII Hospital, Bergamo Italy. New Microbiol..

[30] Buetti N, Tabah A, Loiodice A, Ruckly S, Aslan AT, Montrucchio G, Cortegiani A, Saltoglu N, Kayaaslan B, Aksoy F (2022). Different epidemiology of bloodstream infections in COVID-19 compared to non-COVID-19 critically ill patients: a descriptive analysis of the Eurobact II study. Crit Care.

[31] Pavlaki M, Poulakou G, Drimousis P, Adamis G, Apostolidou E, Gatselis NK, Kritselis I, Mega A, Mylona V, Papatsoris A (2013). Polymicrobial bloodstream infections: epidemiology and impact on mortality. J Glob Antimicrob Resist.

[32] Goldman S, Itshaki O, Shochat T, Gafter-Gvili A, Yahav D, Rubinovitch B, Shepshelovich D (2020). Risk factors and outcome of polymicrobial bacteremia: a retrospective cohort study. Isr Med Assoc J.

[33] Pittet D, Li N, Wenzel RP (1993). Association of secondary and polymicrobial nosocomial bloodstream infections with higher mortality. Eur J Clin Microbiol Infect Dis.

[34] Sancho S, Artero A, Zaragoza R, Camarena JJ, Gonzalez R, Nogueira JM (2012). Impact of nosocomial polymicrobial bloodstream infections on the outcome in critically ill patients. Eur J Clin Microbiol Infect Dis.

[35] Mizusawa M, Carroll KC (2023). Updates on the profile of GenMark's ePlex blood culture identification fungal pathogen panel. Expert Rev Mol Diagn.

[36] Lin K, Zhang HC, Zhao YH, Xia J, Ai JW, Zhang WH (2022). The direct application of plasma droplet digital PCR in the ultra-early pathogen detection and warning during sepsis: case reports. J Infect Public Health.

[37] Berinson B, Both A, Berneking L, Christner M, Lutgehetmann M, Aepfelbacher M, Rohde H (2021). Usefulness of BioFire FilmArray BCID2 for blood culture processing in clinical practice. J Clin Microbiol.

[38] Zboromyrska Y, Cilloniz C, Cobos-Trigueros N, Almela M, Hurtado JC, Vergara A, Mata C, Soriano A, Mensa J, Marco F (2019). Evaluation of the magicplex sepsis real-time test for the rapid diagnosis of bloodstream infections in adults. Front Cell Infect Microbiol.

[39] Yanagihara K, Kitagawa Y, Tomonaga M, Tsukasaki K, Kohno S, Seki M, Sugimoto H, Shimazu T, Tasaki O, Matsushima A (2010). Evaluation of pathogen detection from clinical samples by real-time polymerase chain reaction using a sepsis pathogen DNA detection kit. Crit Care.

