# Supplementary material for: Clinical evaluation of a multiplex droplet digital PCR for pathogen detection in critically ill COVID-19 patients with bloodstream infections
Source: Infection. 2023 Dec 21;52(3):1027–39. doi: 10.1007/s15010-023-02157-x (PMC11143000; doi:10.1007/s15010-023-02157-x)
Supplement: Supplementary file 1 — Supplementary file1 (DOCX 15 kb) [file 15010_2023_2157_MOESM1_ESM.docx]

**Table S1 Completely or partly concordant with all microbiological testing within seven days for** for 76 ddPCR+/BC- and 3 ddPCR+/BC+ but with inconsistent bacteria episodes **.**

| ddPCR positive episodes | Completely concordant | Partly concordant | Inconsistent | Total |
| --- | --- | --- | --- | --- |
| Single Pathogen | 27 | 0 | 17 | 44 |
| Mixed Pathogens | 6 | 24 | 5 | 35 |
| 2 Pathogens | 4 | 11 | 4 | 19 |
| 3 Pathogens | 2 | 7 | 0 | 9 |
| 4 Pathogens | 0 | 4 | 1 | 5 |
| 5 Pathogens | 0 | 1 | 0 | 1 |
| 6 Pathogens | 0 | 1 | 0 | 1 |
| Total | 33 | 24 | 22 | 79 |
