# Supplementary material for: Clinical evaluation of a multiplex droplet digital PCR for pathogen detection in critically ill COVID-19 patients with bloodstream infections
Source: Infection. 2023 Dec 21;52(3):1027–39. doi: 10.1007/s15010-023-02157-x (PMC11143000; doi:10.1007/s15010-023-02157-x)
Supplement: Supplementary file 2 — Supplementary file2 (DOCX 14 kb) [file 15010_2023_2157_MOESM2_ESM.docx]

**Table S2** **177 single positive pathogens identified through ddPCR were compared to pathogens identified from other samples**

| **Bacteria** | **Consistent** | **Inconsistent** |
| --- | --- | --- |
| *A.baumannii* | 40 | 9 |
| *K.pneumoniae* | 22 | 2 |
| *P.aeruginosa* | 9 | 3 |
| *E.coil* | 3 | 5 |
| *S.marcescens* | 4 | 9 |
| *E.cloacae* | 1 | 0 |
| *Enterococcus* | 3 | 12 |
| *Candida* | 13 | 2 |
| *B.fragilis* | 0 | 1 |
| *B.cepacia* | 0 | 2 |
| *CoNS* | 0 | 14 |
| *S.maltophilia* | 0 | 5 |
| *Streptocuccus* | 0 | 18 |
| **Total** | **95** | **82** |
